# Long days restore regular estrous cyclicity in mice lacking circadian rhythms

**DOI:** 10.1016/j.heliyon.2023.e16970

**Published:** 2023-06-02

**Authors:** Takahiro J. Nakamura, Nana N. Takasu, Sayuri Sakazume, Yu Matsumoto, Natsuko Kawano, Julie S. Pendergast, Shin Yamazaki, Wataru Nakamura

**Affiliations:** a Laboratory of Animal Physiology, School of Agriculture, Meiji University, Kawasaki, Kanagawa, 214-8571, Japan; b Department of Oral-Chrono Physiology, Graduate School of Biomedical Sciences, Nagasaki University, Nagasaki, Nagasaki, 852-8588, Japan; c Laboratory of Regulatory Biology, School of Agriculture, Meiji University, Kawasaki, Kanagawa, 214-8571, Japan; d Department of Biology, University of Kentucky, Lexington, KY, 40506, USA; e Department of Neuroscience and Peter O’Donnell Jr. Brain Institute, UT Southwestern Medical Center, Dallas, TX, 75390, USA

**Keywords:** Breeding efficiency, Clock gene, C57BL/6J mice, Photoperiod, Seasonal breeder, Wheel-running

## Abstract

Many female mammals have recurring cycles of ovulation and sexual behaviors that are regulated by reproductive hormones and confer reproductive success. In addition to sexual behaviors, circadian behavioral rhythms of locomotor activity also fluctuate across the estrous cycle in rodents. Moreover, there is a bidirectional relationship between circadian rhythms and estrous cyclicity since mice with disrupted circadian rhythms also have compromised estrous cycles resulting in fewer pregnancies. In the present study, we assessed whether extending day length, which alters circadian rhythms, normalizes estrous cyclicity in mice. We found that *Period* (*Per*) *1*/*2*/*3* triple knockout (KO) mice, that have disabled canonical molecular circadian clocks, have markedly disrupted estrous cycles. Surprisingly, extending the day length by only 2 h per day restored regular 4- or 5-day estrous cycles to *Per1*/*2*/*3* KO mice. Longer days also induced consistent 4-day, rather than 5-day, estrous cycles in wild-type C57BL/6J mice. These data demonstrate that extending daytime light exposure could be used for enhancing reproductive success.

## Highlights

• *Period* mutant mice have highly irregular estrous cycles in 12 h light/12 h dark cycle.
• Extending the day length by only 2 h per day restores regular estrous cycles.
• Long day length also stabilizes the estrous cycle in wild-type mice.

## 1 Introduction

The estrous cycle and circadian rhythms are intricately connected [1]. Developing follicles in the ovaries release estrogens. When peak estrogen levels coincide with a signal from the main circadian pacemaker in the suprachiasmatic nucleus (SCN) on the afternoon of proestrus, there is a surge of luteinizing hormone (LH) that causes ovulation. At the same time, females enter estrus (also known as heat) and become sexually receptive. This interplay between estrogens and the circadian system temporally aligns ovulation with mating and confers reproductive success. Disrupting the circadian system by lesioning the SCN or disabling the function of circadian timekeeping genes (e.g., Clock^Δ19^ mutants, *Per1* KO, *Per2* KO) disrupts this fine-tuning of the estrous cycle with time of day and reduces reproductive success [2].

In mammals, circadian rhythms are generated in cells by a transcriptional-translational feedback loop of circadian gene expression. The circadian genes *Per1*/*2*/*3* are essential components of this molecular timekeeping mechanism since *Per1*/*2*/*3* KO mice lack circadian rhythms when housed in constant conditions (e.g., constant darkness) [3]. However, prior studies showed that light is capable of altering the circadian system in *Per1/2/3* KO mice. Light pulses administered to arrhythmic *Per1/2/3* KO mice in constant darkness induced circadian activity rhythms that persisted for 7 days [3]. Additionally, *Per1/2/3* KO mice have diurnal locomotor activity rhythms in light-dark conditions [4]. Moreover, there is a bidirectional relationship between circadian rhythms and estrous cyclicity since mice with disrupted circadian rhythms also have compromised estrous cycles resulting in fewer pregnancies [5,6]. Thus, we sought to determine whether altering lighting conditions would impact estrous cyclicity in *Per1/2/3* KO females with disrupted circadian rhythms.

## 2 Results and discussion

**Fig. 1:**
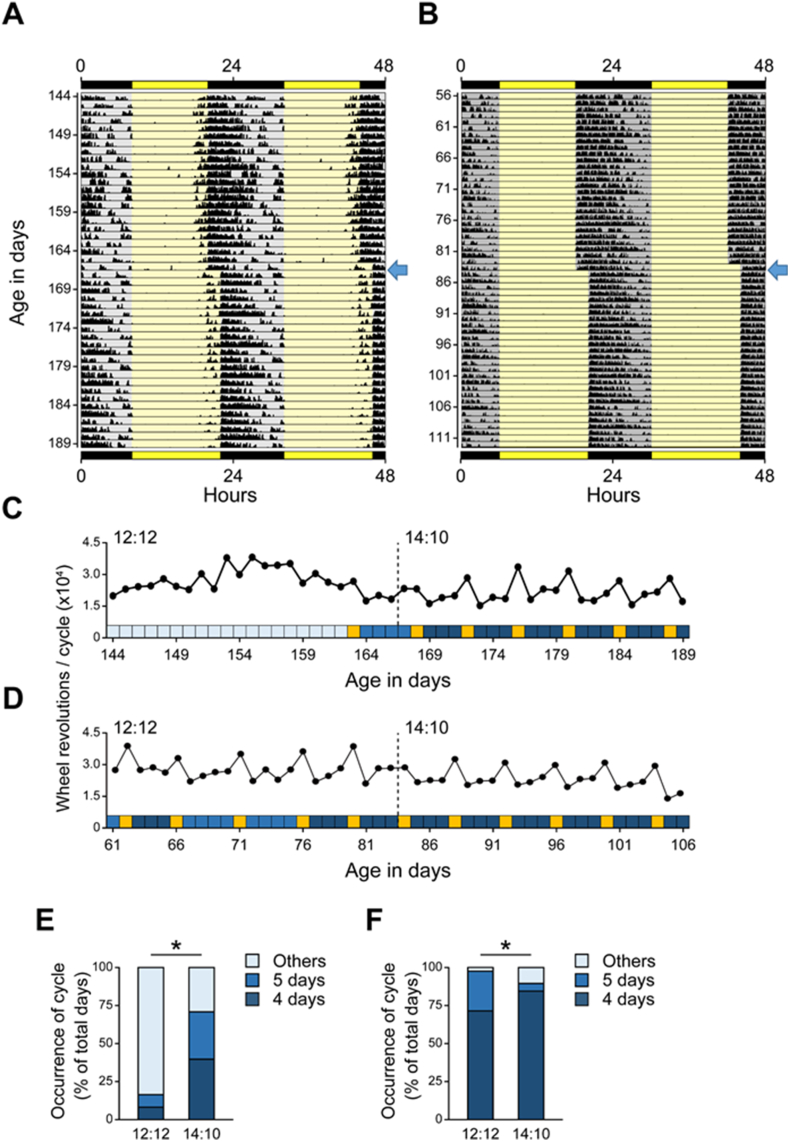
Long days restore regular estrous cyclicity in *Period1/2/3 triple knockout* (*Per1/2/3* KO) and C57BL/6J mice. Female *Per1/2/3* KO (A, C, E) and C57BL/6J (B, D, F) mice were single-housed in standard LD (12 h light:12 h dark) for 28 days then in long-day LD (14 h light:10 h dark) for 28 days. Daily wheel-running activity was continuously measured. Representative double-plotted actograms of wheel-running activity (A, B) and daily numbers of wheel revolutions (C, D) are shown for *Per1/2/3* KO (A, C) and C57BL/6 (B, D) mice. In C and D, the stages of the estrous cycle are shown in colored squares where yellow squares are estrus and dark and medium blue squares show 4-day or 5-day estrous cycles, respectively. Light blue squares represent estrous cycles that are not 4-day or 5-day in duration (other). The ratio of 4-day, 5-day, and other-duration estrous cycles are shown for *Per1/2/3* KO (E) and C57BL/6 (F) mice. *P < 0.001, Pearson’s χ2 test. (For interpretation of the references to color in this figure legend, the reader is referred to the Web version of this article.)

We first studied the estrous cycle in *Per1/2/3* KO females in a 12 h light:12 h dark (12L:12D) cycle. In our previous studies, we showed that the daily wheel-running activity of mice is a proxy for the estrous cycle stage, such that activity is greatest during the night of proestrus/estrus [7]. This approach avoids stress associated with daily handling required for vaginal cytological analysis of the estrous stage. Most *Per1/2/3* KO mice (20 of 21 mice, 95%) did not have regular 4- or 5-day estrous cycles in 12L:12D (Fig. 1A and C). The duration of the estrous cycle in *Per1/2/3* KO mice was highly variable, and some mice entered estrus only once or twice during the 28 days of the experiment (Figs. S1 and S3).

We next investigated whether altering light exposure, by lengthening the day by 2 h, affected estrous cycles in *Per1/2/3* KO mice. When *Per1/2/3* KO mice were transferred to longer days (14L:10D), 38% (8 of 21) of mice had 4- or 5-day estrous cycles (Fig. 1A and C). This marked increase in regular estrous cyclicity in longer days was unexpected (Fig. 1E, *P* < 0.001, Pearson’s χ^2^ test). Notably, C57BL/6J mice, which is the background strain of the *Per1/2/3* KO mice, do not produce pineal melatonin which is known to mediate photoperiodic responses in rodents [8]. Therefore, we ruled out a role for melatonin in regulating the improvement in estrous cyclicity in our study.

Most strains of laboratory mice are not considered seasonal breeders because changes in day length do not induce physiological responses related to reproductive functions and body weight [9]. Nonetheless, The Jackson Laboratory maintains its breeding colonies of non-seasonal mice at 14L:10D to extend the hours when employees can access the mice (communication with The Jackson Laboratory). Thus, we next determined whether longer days affected the estrous cycle in wild-type C57BL/6J mice. In 12L:12D, 47% of C57BL/6J mice (9 of 19) consistently had 4-day estrous cycles, while the rest showed 4-day estrous cycles mixed with 5-day or other cycles (Fig. 1B and D, S2, S4, and S5). After lengthening the day to 14L:10D, 68% of C57BL/6J mice (13 of 19) had continuous 4-day estrous cycles (Fig. 1F, *P* < 0.001, Pearson’s χ^2^ test).

**Fig. 2:**
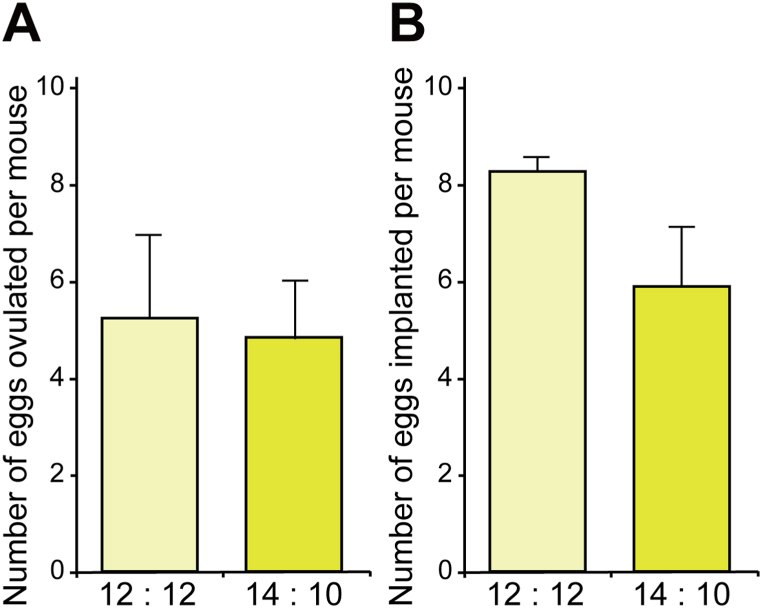
Long-day LD cycles do not influence the frequency of ovulation and implantation in C57BL/6J mice. (A, B) The number of eggs ovulated (A) and implanted (B) per mouse housed in the standard LD cycle (12 h light:12 h dark) or long-day LD (14 h light:10 h dark) cycle. Data are represented as mean ± SEM (n = 8 each for ovulation; n = 8 each for implantation).

If the estrous cycle shortens from 5 days to 4 days, the probability of pregnancy increases from once every 5 days to once every 4 days. We confirmed that wild-type females had eggs in their oviducts on the day of estrus. Thus, it is possible that the shortened 4-days estrous cycle in long days could improve reproductive success if the number of eggs ovulated or the number of eggs implanted in the uterus per cycle is unchanged. Therefore, we next assessed whether the longer day length influenced the number of eggs and implantations. There was no significant effect of day length on the number of eggs that were ovulated (Fig. 2A) and implanted (Fig. 2B). Thus wild-type mice in long days had the same number of eggs ovulated and implanted, but ovulation likely occurred more frequently. These results suggest that lengthening the day by 2 h could increase reproductive success by increasing the frequency of ovulations.

Together, we found that simply lengthening the day by 2 h markedly improved estrous cyclicity in both wild-type C57BL/6J mice and in mice with severe circadian disruption. Regarding the potential mechanism, we hypothesize that the amplitude of the light-driven SCN of *Per1/2/3* triple KO mice is not robust enough to precisely gate the estrous cycle. Lengthening the day length to 14 h enhances the SCN amplitude and this provides a more salient gate which confers a stably rhythmic estrous cycle in *Per1/2/3* triple KO mice. The longer 14 h day may also enhance the amplitude of the wild-type SCN in 14L:10D which could enhance the stability of the gating signal and/or the phase or duration of the gate during which the LH surge can occur. Future investigation of SCN rhythms in wild-type and *Per1/2/3* SCN rhythmicity under 12L:12D and 14L:10D photoperiods may inform the mechanisms that regulate long-day regulation of the estrous cycle. Historically, changes in the duration of the daily melatonin rhythm by photoperiod explains photoperiodic responses in animals. However, the C57BL/6J mouse strain which we used in the current study is melatonin deficient. Further studies of photoperiodic regulation of the duration and stability of the estrous cycle in melatonin deficient mice will provide mechanistic insights into photoperiodism in mammals.

Like laboratory mice, humans are not categorized as seasonal breeders. However, seasonal fluctuations in human fertility have been reported [10]. This study suggests that while “non-seasonal” mammals may not have overt reproductive responses, such as gonadal recrudescence, to changes in day length, they may still retain subtle reproductive responses that can nonetheless affect reproduction.

In conclusion, this study demonstrates that long-day photoperiod could enhance reproductive success by improving the regularity of estrous cyclicity in both circadian mutant and wild-type mice. Future studies should investigate whether long-day regulation of estrous cyclicity translates into more frequent ovulations and improved reproductive success. If so, then environmental lighting conditions could be a therapeutic target for improving reproductive success.

## 3 Materials and methods

### 3.1 Ethical statement

All procedures and standards of care were conducted according to the guidelines of the Japanese Physiological Society and approved by the Institutional Animal Care and Use Committee at the School of Agriculture, Meiji University (approval number: IACUC16-0012) and the Graduate School of Biomedical Sciences, Nagasaki University (approval number:1809181479-2).

### 3.2 Animals and housing

Female C57BL/6J mice were purchased from Japan SLC (Shizuoka, Japan). *Per1*^*−/−*^*/Per2*^*−/−*^*/Per3*^*−/−*^; KO (*Per1*/*2*/*3* KO; mice congenic with the C57BL/6J strain) were generated as previously described [4]. Genotyping was carried out by PCR using two sets of primers that amplified the wild-type or disrupted gene for each of the *Period* genes [11,12]. Animals were bred and maintained in a standard 12 h light and 12 h dark cycle (room temperature, 23 ± 1 °C; humidity, 50 ± 10%). Food (Labo MR Stock, NOSAN corp., Yokohama, Japan, or MF, Oriental Yeast Co Ltd., Tokyo, Japan) and water were provided *ad libitum*.

### 3.3 Measuring wheel-running activity

Each mouse was individually housed in a cage (183 × 340 × 148 mm; CL-0135, CLEA Japan, Tokyo Japan) equipped with a running wheel (12 cm diameter, Sanko, Osaka, Japan). Only female mice were housed in light-tight boxes (no males were present). The cages were placed in light-tight, ventilated boxes, and the light intensity at the bottom of the cage was 100–300 lux (white light LED LDA2L-GAG5, Ohm Electric Inc., Saitama, Japan). The number of wheel revolutions was counted by a magnetic sensor-activated signal (59070-010, Littelfuse, Inc., Chicago, IL, USA) by a button magnet on the running wheel and recorded by a computer every minute. The data were analyzed by the Chronobiology Kit (Stanford Software Systems, Naalehu, HI, USA) and ClockLab software (Actimetrics, Wilmette, IL). The actograms were plotted in the scaled format.

### 3.4 Determination of estrous cycle length

The stages of the estrous cycle were determined by the pattern of daily total activity changes as previously described [7,13,14]. Briefly, proestrus/estrus was defined as the day when there was a peak in total wheel revolutions that was preceded and succeeded by at least 2–3 days of lower activity. We defined this peak as “estrus” since previous studies demonstrated that wheel-running activity is synchronized with the ovulatory cycle and animals run more on the nights of proestrus than other diestrus nights [[15], [16], [17], [18], [19], [20]]. Occasionally activity levels peaked for 2 days before declining to a trough. In this case, the 2nd day of peak activity was defined as estrus. Adult C57BL/6J mice have a 4-day or 5-day estrous cycle [7,14]. Therefore, we defined 4-day or 5-day estrous cycles as regular estrous cycles. If the estrous cycle appeared shorter than 4 days or longer than 5 days, this was classified as other (irregular). Among the recording, the percentage of days in each cycle duration (4-day, 5-day, or other) were determined for each mouse between the first day estrus was observed and the last day estrus was observed. Then the percentage of days spent in each group (4-day, 5-day, or other) were compiled for all mice of each genotype in 12L:12D and 14L:10D (Fig. 1E and F, and S5E).

### 3.5 Vaginal smear test for the experiment of ovulation and implantation

The stage of the estrous cycle was determined using vaginal cytology as previously described [21]. Briefly, a vaginal smear was collected with a moistened cotton swab, applied to a glass slide, stained with hematoxylin, and then observed under a stereomicroscope. According to the microscopic characteristics of the vaginal smear, the mice were classified as proestrus, estrus, metestrus, or diestrus.

### 3.6 Evaluation of ovulated eggs

Eggs were collected from the oviductal ampulla of female mice staged at estrus to analyze natural ovulation. The total number of eggs that were ovulated at estrus were counted for each mouse.

### 3.7 Evaluation of implantation

Female mice staged at proestrus were housed with age matched C57BL/6J male mice (Japan SLC, Shizuoka, Japan). Female mice with vaginal plugs the next morning were determined to be pregnant, and decidua was observed at the implant site 1 week after copulation.

### 3.8 Statistics

Results were considered significant at *P* < 0.05. Pearson’s χ2 test was used to compare the estrous cycle duration between the two groups. For other data, Student’s *t*-tests were used to examine the differences between the two groups.

### 3.9 Experimental design

#### 3.9.1 Effects of long days on estrous cyclicity

Female *Per1/2/3* KO (109 ± 32 days old, n = 21) and wild-type C57BL/6J (49 days old, n = 19) were individually housed in cages with running wheels. The wheel-running activity of the mice was recorded in the standard LD cycle (12 h light: 12 h dark) for 28 days and then in the long-day LD cycle (14 h light: 10 h dark) for 28 days. In another cohort, wild-type female C57BL/6J mice (49 days old, n = 10) were individually housed in cages with running wheels. The wheel-running activity of the mice was recorded in the standard LD cycle (12 h light: 12 h dark) for 100 days.

#### 3.9.2 Effects of long days on the frequency of ovulation and implantation

To test the ovarian function of wild-type mice in two different photoperiods, seven-week-old female C57BL/6J mice were housed in either standard LD (n = 16) or long-day LD (n = 16). Vaginal smears were collected daily until 12 weeks old, or ovulation (n = 8) and implantation (n = 8) were examined.

## Author contribution statement

Takahiro J. Nakamura and Wataru Nakamura: Conceived and designed the experiments; Performed the experiments; Analyzed and interpreted the data; Contributed reagents, materials, analysis tools or data; Wrote the paper.

Nana N. Takasu and Yu Matsumoto: Performed the experiments.

Sayuri Sakazume: Performed the experiments; Analyzed and interpreted the data.

Natsuko Kawano: Conceived and designed the experiments; Analyzed and interpreted the data; Wrote the paper.

Julie S. Pendergast and Shin Yamazaki: Analyzed and interpreted the data; Wrote the paper.

## Data availability statement

All data for data analysis are available at Mendeley data (Reserved https://doi.org/10.17632/33tbmnspd7.2).

## Declaration of competing interest

The authors declare that they have no known competing financial interests or personal relationships that could have appeared to influence the work reported in this paper
