# Supplementary material for: Long days restore regular estrous cyclicity in mice lacking circadian rhythms
Source: Heliyon. 2023 Jun 2;9(6):e16970. doi: 10.1016/j.heliyon.2023.e16970 (PMC10361014; doi:10.1016/j.heliyon.2023.e16970)
Supplement: Multimedia component 1 [file mmc1.docx]

**Supplemental Information**

**Long days restore regular estrous cyclicity in mice lacking circadian rhythms**

Takahiro J. Nakamura, Nana N. Takasu, Sayuri Sakazume, Yu Matsumoto, Natsuko Kawano, Julie S. Pendergast, Shin Yamazaki, and Wataru Nakamura

**Supplemental Figures**


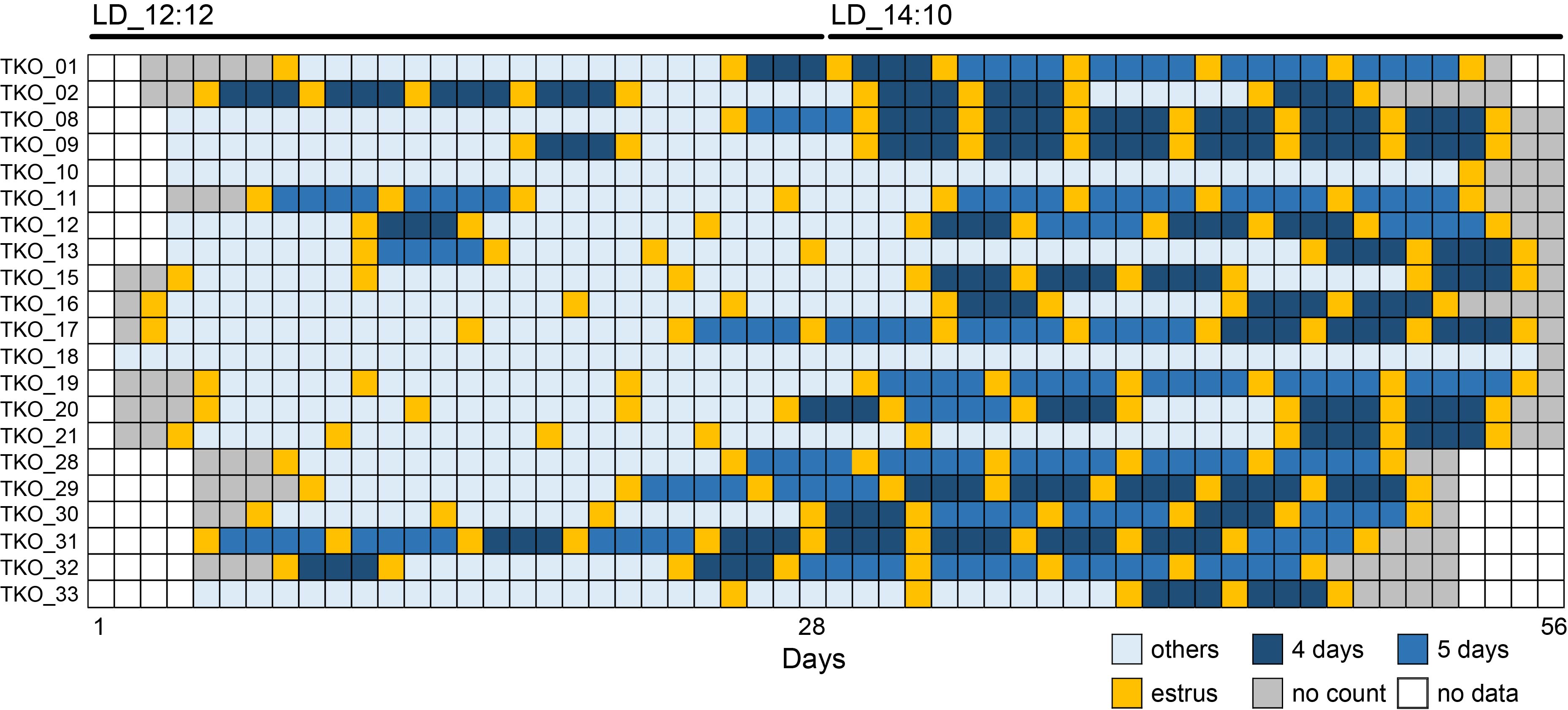


**Figure S1. Estrous cycles of individual *Per1/2/3* KO mice under long-day LD cycle.**

Estrous cycles of all *Per1/2/3* KO mice under long-day LD cycle that were recorded in the present study were shown (n=21). The stages of the estrous cycle are shown in colored squares where yellow squares are estrus and dark and medium blue squares show 4-day or 5-day estrous cycles, respectively. Light blue squares represent estrous cycles that are not 4-day or 5-day in duration (other). Gray squares indicate cycle length cannot be determined due to no information of previous or after estrus.


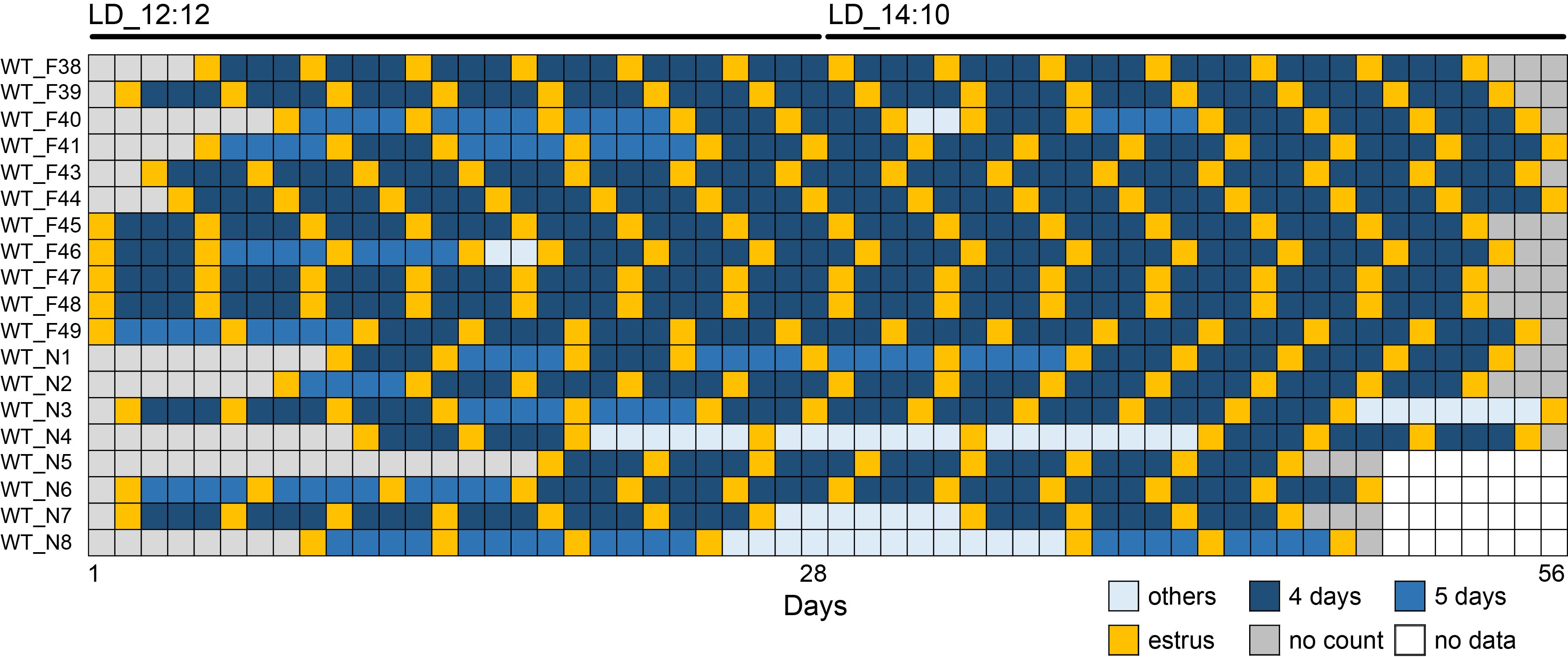


**Figure S2. Estrous cycles of individual C57BL/6J mice under long-day LD cycle**

Estrous cycles of all C57BL/6J mice under long-day LD cycle that were recorded in the present study were shown (n=19). The stages of the estrous cycle are shown in colored squares where yellow squares are estrus and dark and medium blue squares show 4-day or 5-day estrous cycles, respectively. Light blue squares represent estrous cycles that are not 4-day or 5-daysin duration (other). Gray squares indicate cycle length cannot be determined due to no information of previous or after estrus.


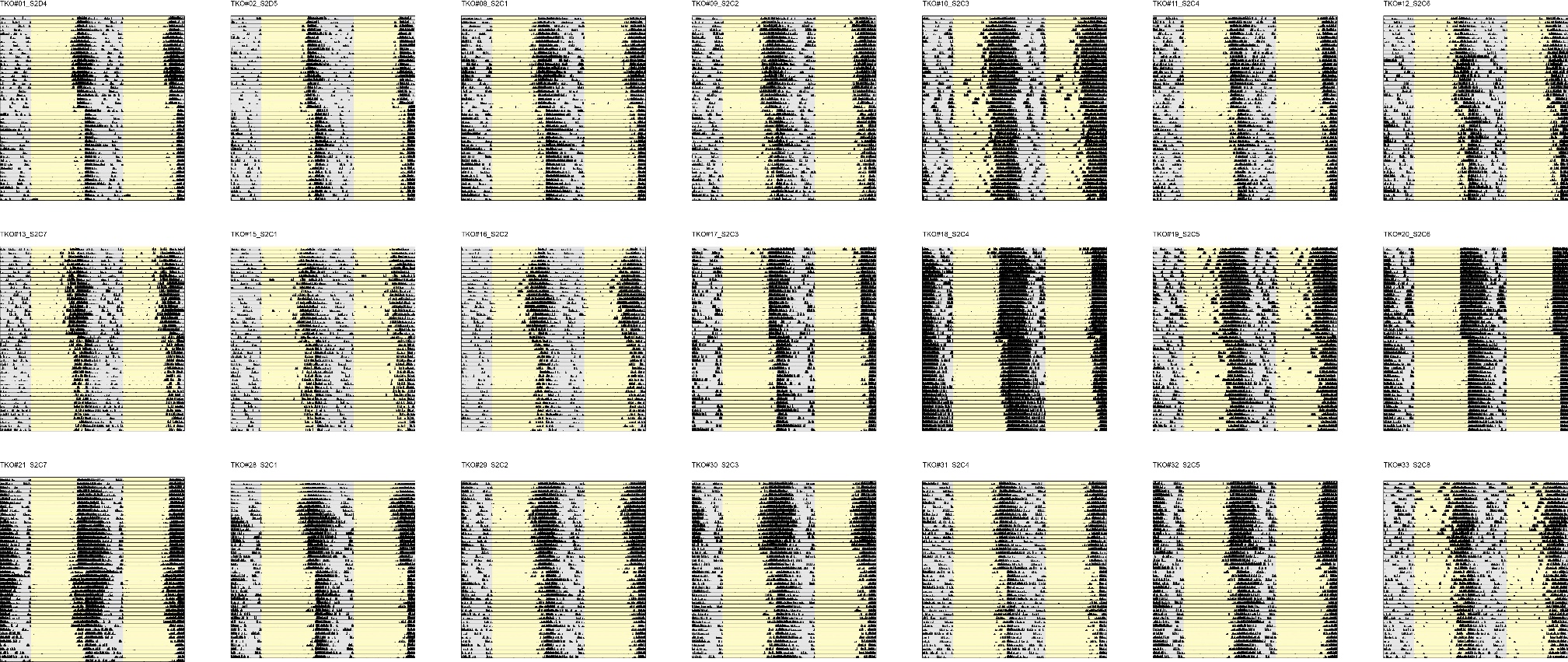
**Figure S3. Actograms of all individual *Per1/2/3* KO mice** **under long-day LD cycle that were recorded in the present study (n=21)**


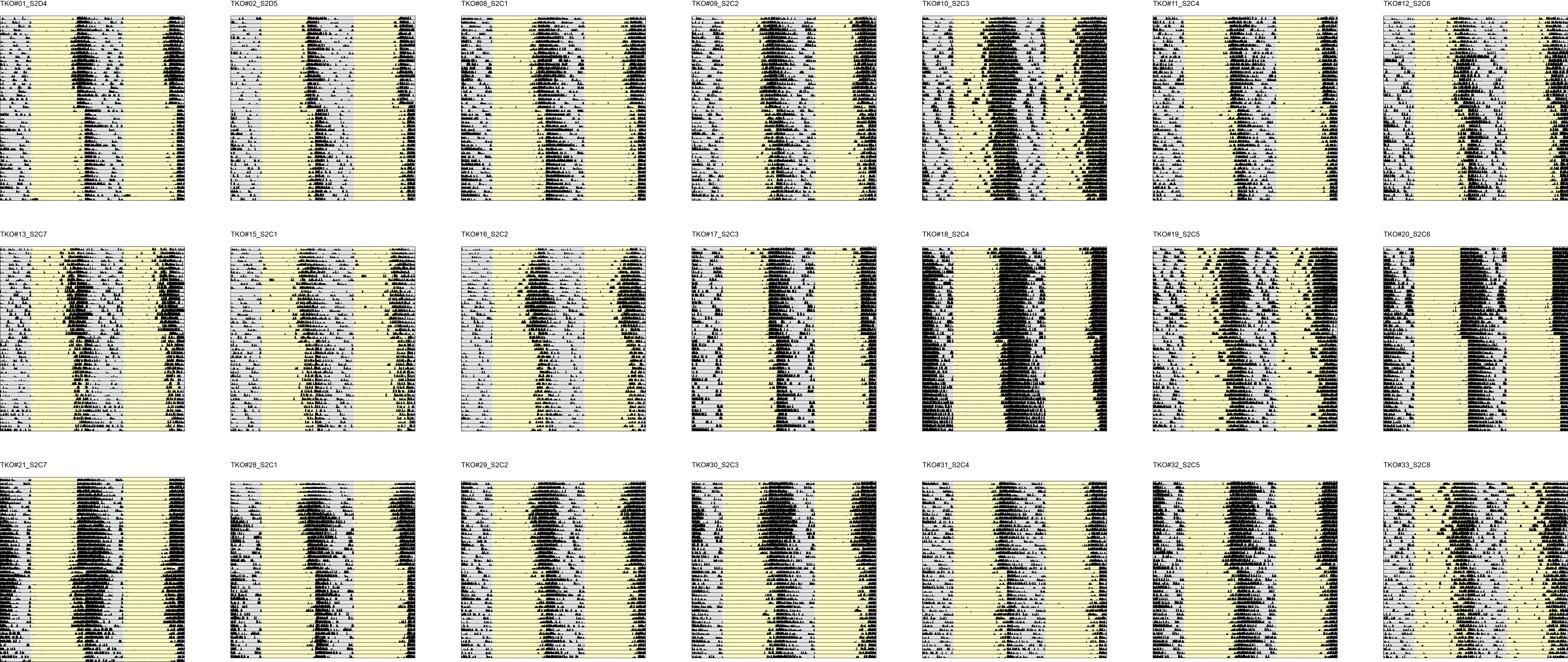


**
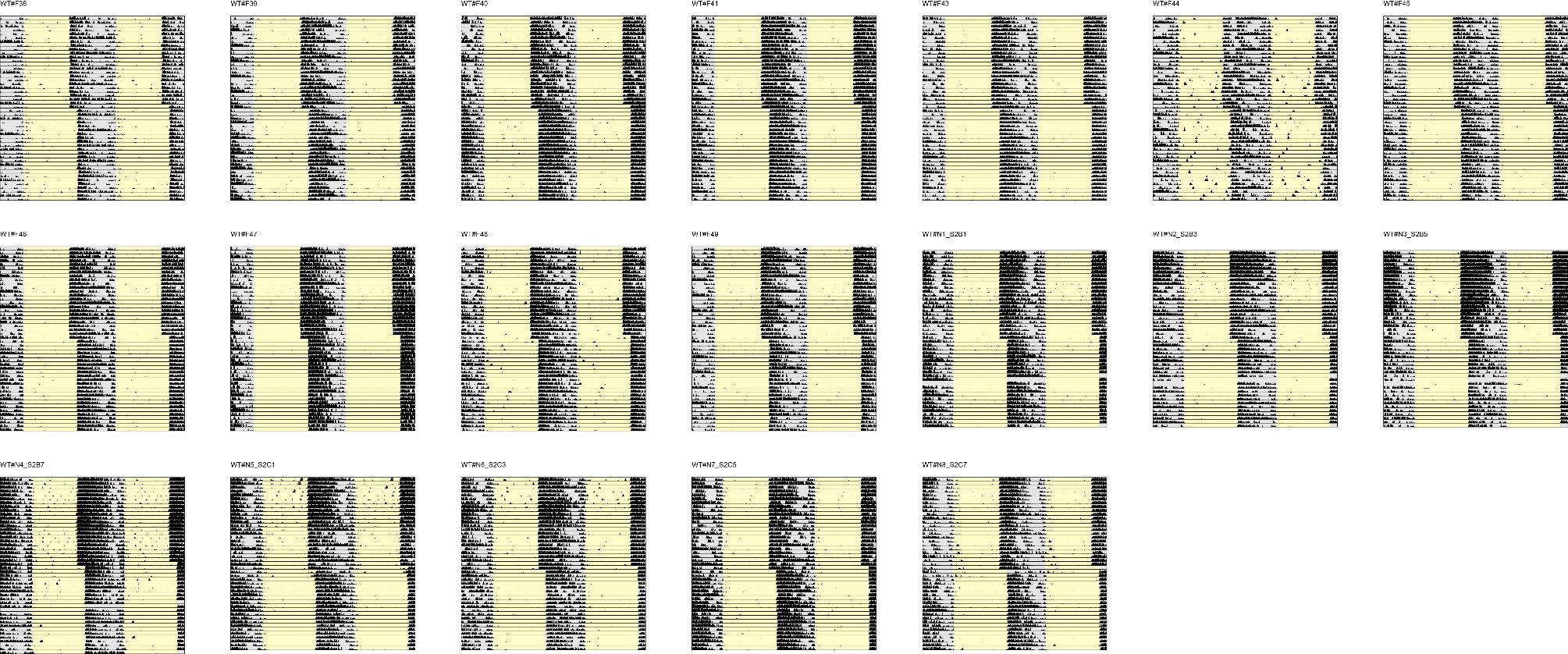
Figure S4. Actograms of all individual wild-type mice under long-day LD cycle that were recorded in the present study (n=19)**


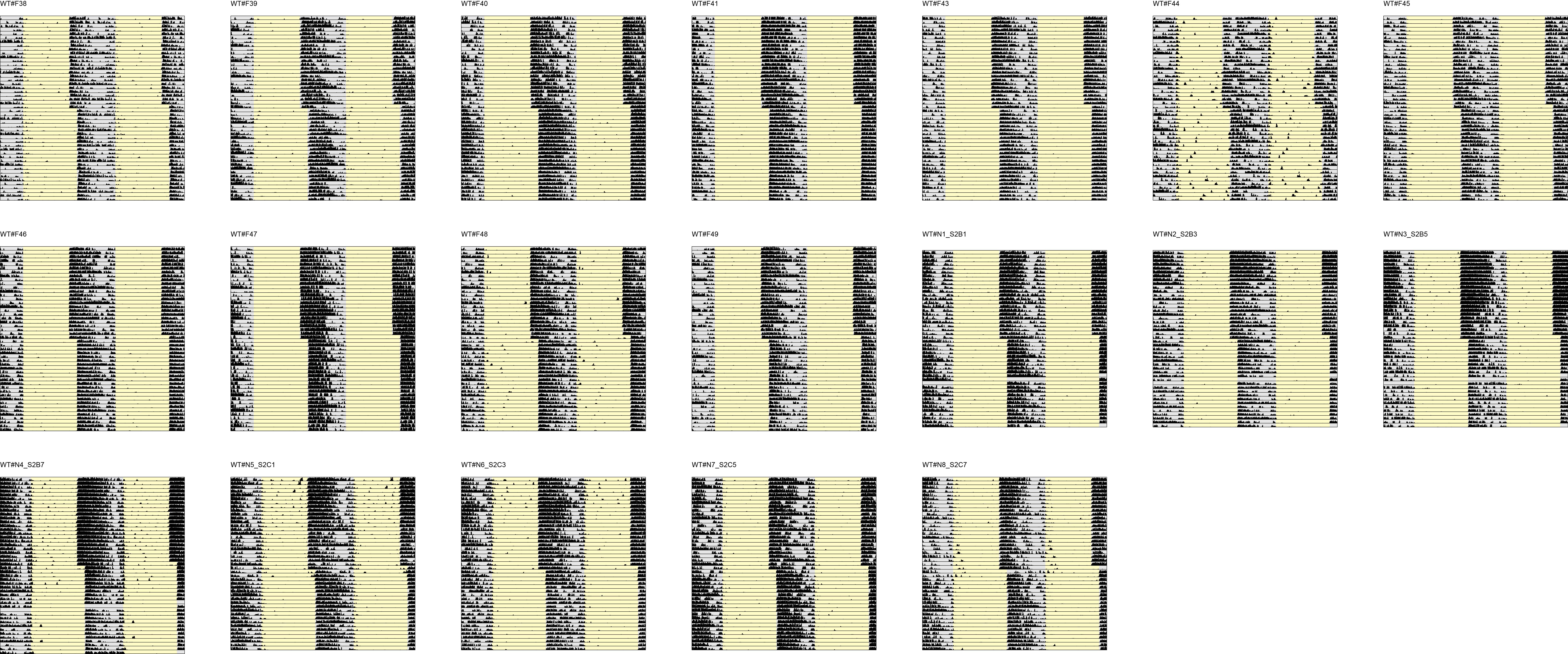

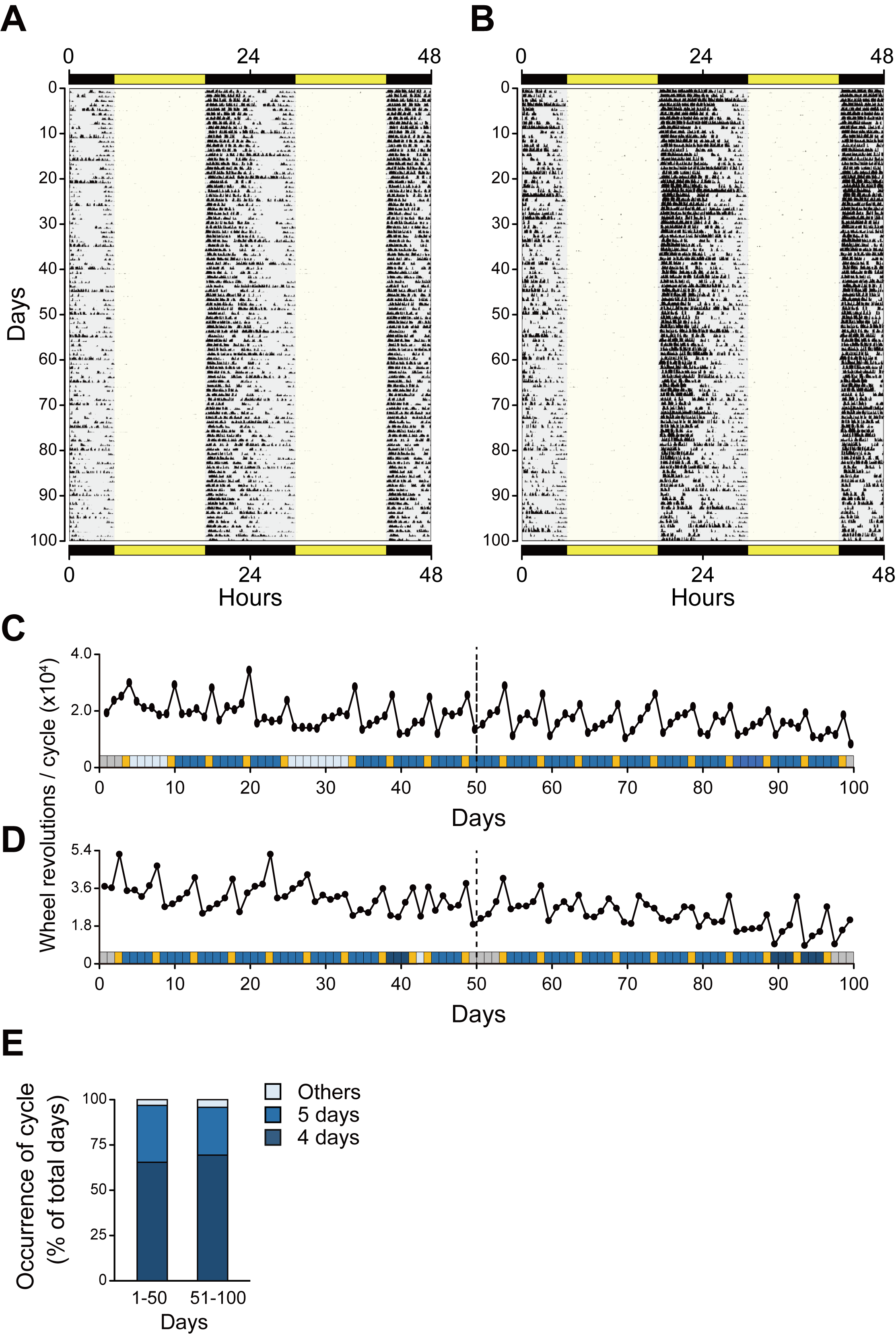


**Figure S5. Effect of continuous standard LD cycle on the cycle length of estrous cycle in C57BL/6J mice.**

We investigated the effect of continuous standard LD (12 hours light:12 hours dark) on the cycle length of the estrous cycle in C57BL/6J mice. Seven-week-old female C57BL/6J mice were single-housed in standard LD (12 hours light:12 hours dark) for 100 days. The daily wheel-running activity was continuously measured. Two representative double-plotted actograms of wheel-running activity (A, B) and daily numbers of wheel revolutions (C, D) are shown. In C and D, the stages of the estrous cycle are shown in colored squares where yellow squares are estrus and dark and medium blue squares show 4-day or 5-day estrous cycles, respectively. Light blue squares represent estrous cycles that are not 4-day- or 5-day in duration (other). (E) The ratio of 4-day, 5-day, and other-duration estrous cycles is divided into the first 50 days (day 1 – 50) and last 50 days (day 51 – 100) and compared. In the first 50 days, mice (n=10) exhibited 62.4% of 4-day, 33.9% of 5-day, and 3.6% of other-day estrous cycle. In the last 50 days, 66.1% of 4-day, 28.3% of 5-day, and 5.7% of other-day estrous cycle were observed. No statistical difference was detected between the two groups (*P*>0.05, Pearson’s χ2 test). The result indicates that maturity or aging does not influence the appearance of the 4-day or 5-day estrous cycle.


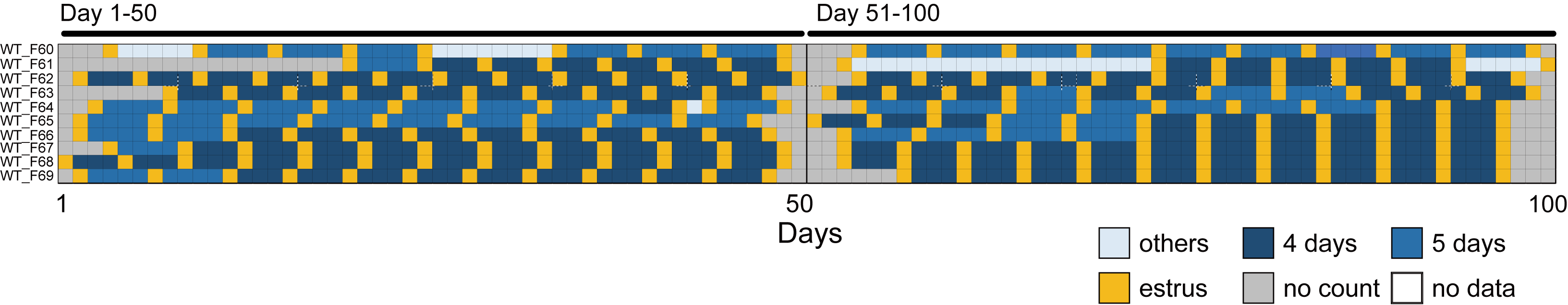


**Figure S4. Estrous cycles of individual C57BL/6J mice under continuous standard LD cycle**

Estrous cycles of all C57BL/6J mice under continuous standard LD cycles that were recorded in the present study were shown (n=10). The stages of the estrous cycle are shown in colored squares where yellow squares are estrus and dark and medium blue squares show 4-day or 5-day estrous cycles, respectively. Light blue squares represent estrous cycles that are not 4- or 5-days in duration (other). Gray squares indicate cycle length cannot be determined due to no information of previous or after estrus.
